# What Are the Causes of the Venous Circulation?

**Published:** 1875-10

**Authors:** J. G. Knox


### WHAT ARE THE CAUSES OF THE VENOUS CIRCULATION?

BY J. G. KNOX, A. B., M. D.

The veins, like the arteries, are composed of three coats, an inner, middle and exterior. But they contain a less quantity of muscular and elastic fibres, and a larger proportion of simple condensed areolar tissue. They are, therefore, less elastic, contractile and more flaccid and compressible than the arteries. The veins of the limbs, neck, and external portions of the head and trunk are also provided with valves which allow the blood to pass readily from the capillaries towards the heart, but effectually prevent its reflux in the opposite direction. The veins are deficient in that elasticity and resiliency which characterize the arteries; consequently, when filled with blood, they enlarge to a certain extent and collapse when the pressure is removed; whereas the arteries, when cut across and emptied of blood, remain open and pervious, still retaining the tubular form. Another peculiarity of the venous system is the great abundance of veins and their inosculations with each other, thus furnishing so many channels through which the blood can flow from the periphery toward the heart. The question now arises, why does the blood return from the periphery to the heart? What are the physical or physiological forces which impel the blood to flow from the capillaries to the heart through this vast number of channels, composed of flaccid and inelastic walls with their numerous inosculations, producing currents having every degree of angle with each other up to a right angle? And there is another still greater obstacle to be overcome, viz: the force of gravity. Let us examine the causes assigned by physiologists, and see if they are sufficient to overcome all these impediments and cause the blood to flow through the veins from the feet to the heart while a man is in the erect position.

Professor Dalton, and I believe almost all other physiologists, assign three causes: 1. The respiratory movements. 2. The contraction of the voluntary muscles. 3. The force of the capillary circulation. Let us examine the first cause. They say "that when the chest expands, lifting the ribs and forcing the diaphragm down, that these movements diminish the pressure exerted upon its contents, and so have the effect of drawing into the chest all the fluids which can gain access to it." They say "that its influence is extended to the farthest extremities of the venous system, the blood being gently solicited towards the heart at each expansion of the chest." This cause would appear more plausible if the act of inspiration was synchronous with the diastate of the heart, and the act of expiration with the systate of the heart. But we know such is not the case, and all physiologists admit that the expiratory act has exactly the opposite effect of the act of inspiration, and, therefore, must, to a very great extent, neutralize it. It seems that rather too much importance has been attached to this force, if force it really be, even if it was not neutralized as shown above. At the end of a full inspiration, are not the lungs inflated with air, and is not this the cause of the expansion of the walls of the chest and the descent of the diaphragm? If so, the chest is full of air, and there is in fact no vacuum formed in it to draw or solicit the blood in the veins to the heart. Second. It is contended that as the veins lie among the voluntary muscles, the alternate contraction and relaxation of these helped to drive the blood along in the veins. That these may have some little influence under certain circumstances will not be denied, but may they not very frequently interfere with and retard it? The circulation is never carried on more easily and regularly than when a person is in a quiet sleep, when there is not a movement of any of the voluntary muscles. And in cases of paralysis, when it is impossible for the patient to move a single muscle in the paralyzed limb, yet the circulation goes on, not quite as vigorously, it is true, as in the limb not affected, yet all the blood sent out through the arteries returns through the veins. So that the contraction and relaxation of the voluntary muscles do not appear to be absolutely necessary to the circulation of the blood in the veins. We come now to the consideration of the third cause, namely, the force of the capillary circulation. Professor Dalton, in his admirable work, says: "This is the most important of all, as it is the only one which is constantly and universally active." He says: "The circulation going on constantly in the capillaries, everywhere tends to crowd the radicles of the veins with blood, and this vis a tergo or pressure from behind fills the whole venous system by a constant and steady accumulation." In order that we may analyze this force, let us study the structure, size and circulation of the capillaries. They are composed, then, according to Professor Dalton, "of a simple, homogeneous, nucutated, tubular membrane, which is continuous with the internal arterial tunic. They contain neither the external and fibrous tunic, nor the middle or muscular coat of the arteries. Their average size in the human subject is a little over 1-3000 of an inch, and they inosculate with each other in every direction, and thus form an interlacing net-work." And he says: "The blood passes in a uniform current without any apparent contraction or dilitation of the vessels, very much as if it were flowing through glass tubes. The numerous streams do not tend toward any one particular point. They pass above and below each other at right angles to each other's course, or even in opposite directions, moving at the rate of 1-30 of an inch per second." Now can the blood, moving at this slow rate through these very minute vessels, devoid of muscular tissue, running in every direction, inosculating freely with each other at every degree of angle, act as a vis a tergo to impel the blood through the flaccid and inelastic veins, and this, too, often in opposition to the laws of gravity? It is very easily understood how the blood is forced through the arteries by the contraction of the heart, for here we have a cause fully adequate to produce the effect. But it is not so readily comprehended how any or all of the causes, assigned by physiologists, can cause the return of the blood through the veins to the heart. It does seem that they have entirely ignored the most powerful and effective cause, to-wit, the vacuum formed in the heart by the act of dilitation. The contraction of the heart begins with the auricles, forcing all the blood in them into the ventricles, which next contract, forcing the blood out of them through the aorta and pulmonary artery. This, you will observe, is a continuation of the contraction begun in the auricles; and when completed, there is no blood in either auricles or ventricles, and then the dilitation takes place, which of course forms a vacuum in both auricles and ventricles, and the pressure of the atmosphere on every part of the body, internal as well as external, causes the blood to rush back through the veins to the heart to fill the vacuum thus formed. There is an old but very true adage, that "Nature abhors a vacuum." And this explains why it is so fatal to admit air into the veins; in the operation of transfusion, the air rushes on ahead of the blood and fills the vacuum, and thus effectually puts a stop to the circulation. The blood being no longer invited, or drawn forward to the heart by reason of a vacuum, but its entrance is resisted by the elastic force of the air already expanded by the warmth of the blood and the internal heat of the body, so that it fills the vacuum and effectually excludes the blood from the heart. This seems to be the easiest and most natural way to account for the venous circulation, and we have in this an adequate force to accomplish the work without having recourse to the causes usually assigned, although they may aid to a limited extent. The velocity with which the blood moves in the different parts of its circuit from the time it leaves the heart, through the arteries, till it arrives at the heart again, through the veins, goes to prove that a vacuum formed in the heart, at each dilitation, is the main cause of the venous circulation. All physiologists agree that the velocity in the aorta, near the heart, is much greater than in the arteries further removed from it, and that its velocity becomes less and less until it reaches the capillaries, when it drops down to one or two inches per minute. This is strictly in accordance with the laws which govern bodies sent forward by a projectile force, or a vis a tergo through the air, or any other medium, or through elastic tubes. They, meeting with resistance in their passage through the air, become slower and slower, until they cease to move forward, and fall to the ground. So the blood meets with mechanical obstacles in the friction against the walls of the vessels, and the friction caused by the division of the arteries into numerous branches at every degree of angle, thus diminishing in size and increasing in number until they are merged into the capillaries. And thus the velocity is gradually reduced from twenty inches per second to a little over one inch per minute in the capillaries. But the law of projectile forces does not apply to the return of the blood from the capillaries to the heart, as it moves very slowly at first and gradually increases till it reaches the heart. It cannot be a vis a tergo that drives it forward, then, but a vis in fronté, which draws it forward nearer in accordance with the laws of attraction. For instance, if you drop a ball from the top of a high tower, it will move slowly through the first foot, but its velocity will increase continually till it reaches the ground. Thus the vacuum formed in the heart invites or draws the blood to it through the influence of atmospheric pressure, and its velocity increases gradually from the time it leaves the capillaries till it arrives at the auricles.

You see, then, that the blood in the veins returns to the heart in strict accordance with the laws of attraction. It leaves the capillaries with a velocity of between one and two inches per minute, and when it reaches the heart it has acquired a velocity of about fourteen or fifteen inches per second. The momentum acquired by any moving body, or fluid, is equal to the product of the quantity of matter into the velocity. The small quantity of fluid in one of these very minute capillaries into the very slow velocity will not give much momentum. They are very numerous, it is true, but their currents are in every direction, so as in a great measure to neutralize each other's momentum. We know that the blood is sent out from the heart through the arteries with a powerful force, yet the resistance it meets with continually diminishes its velocity until it reaches the capillaries, although the arteries offer far less resistance than the more numerous and flaccid veins. And it is difficult to see why the blood in the veins should not be governed by the same law if moved by a force from behind.

If the theory, that the capillary circulation propels the blood through the veins to the heart, be true, the force should be greater than that of the heart, and the blood should leave the capillaries with a velocity of fourteen or fifteen inches per second, and after overcoming all the impediments, such as thin flaccid veins, numerous inosculations, and the force of gravity, reach the heart with a velocity about equal to that of the capillary circulation. That is, for that theory to be consistent with the laws of nature, the velocity of the blood should continually diminish from the time it leaves the capillaries till it reaches the heart. But we know such is not the case; therefore, that theory cannot be true.

